# The knowns and unknowns of neural adaptations to resistance training

**DOI:** 10.1007/s00421-020-04567-3

**Published:** 2020-12-23

**Authors:** Jakob Škarabot, Callum G. Brownstein, Andrea Casolo, Alessandro Del Vecchio, Paul Ansdell

**Affiliations:** 1 School of Sport, Exercise and Health Sciences, Loughborough University, Loughborough, UK; 2 Laboratoire Interuniversitaire de Biologie de la Motricité, Université Jean Monnet Saint-Etienne, Université Lyon, Saint-Étienne, France; 3 Department of Bioengineering, Imperial College London, London, UK; 4 Department of Biomedical Sciences, University of Padova, Padua, Italy; 5 Department of Artificial Intelligence and Biomedical Engineering, Faculty of Engineering, Friedrich-Alexander University, Erlangen-Nurnberg, 91052 Erlangen, Germany; 6 Department of Sport, Exercise and Rehabilitation, Faculty of Health and Life Sciences, Northumbria University, Newcastle upon Tyne, NE1 8ST UK

**Keywords:** Descending tracts, High-density surface electromyography, Motor cortex, Motor neuron, Strength, Synaptic input, Transcranial magnetic stimulation

## Abstract

The initial increases in force production with resistance training are thought to be primarily underpinned by neural adaptations. This notion is firmly supported by evidence displaying motor unit adaptations following resistance training; however, the precise locus of neural adaptation remains elusive. The purpose of this review is to clarify and critically discuss the literature concerning the site(s) of putative neural adaptations to short-term resistance training. The proliferation of studies employing non-invasive stimulation techniques to investigate evoked responses have yielded variable results, but generally support the notion that resistance training alters intracortical inhibition. Nevertheless, methodological inconsistencies and the limitations of techniques, e.g. limited relation to behavioural outcomes and the inability to measure volitional muscle activity, preclude firm conclusions. Much of the literature has focused on the corticospinal tract; however, preliminary research in non-human primates suggests reticulospinal tract is a potential substrate for neural adaptations to resistance training, though human data is lacking due to methodological constraints. Recent advances in technology have provided substantial evidence of adaptations within a large motor unit population following resistance training. However, their activity represents the transformation of afferent and efferent inputs, making it challenging to establish the source of adaptation. Whilst much has been learned about the nature of neural adaptations to resistance training, the puzzle remains to be solved. Additional analyses of motoneuron firing during different training regimes or coupling with other methodologies (e.g., electroencephalography) may facilitate the estimation of the site(s) of neural adaptations to resistance training in the future.

## Introduction

Resistance exercise is one of the most common exercise modalities, providing numerous functional and physiological benefits to various populations, from athletes to patients. Following a period of resistance training, the maximal volitional force generating capacity of skeletal muscles is typically increased. Though long-term resistance training is accompanied by modifications of muscle morphology, the initial (< 2–4 weeks) increases in force production are thought to be primarily underpinned by neural adaptations (Moritani and DeVries 1979; for reviews see Enoka 1988; Sale 1988; Carroll et al. 2001; Folland and Williams 2007). The existence of neural adaptations is purported due to several behavioural observations such as task-specificity of strength in the absence of significant morphological adaptations (e.g., Ansdell et al. 2020), the disproportionate increase in muscle force relative to muscle size (Moritani and DeVries 1979; Häkkinen et al. 1998) and the increase in voluntary activation (Lee et al. 2009) in the initial weeks of training, the phenomenon of cross-education, whereby unilateral resistance training of one limb increases force production of contralateral homologous muscle group (Manca et al. 2018), and increased muscle force generating capacity following weeks of imagined contractions (e.g., Zijdewind et al. 2003).

Whilst there is a paucity of methods allowing direct assessment of neural activity in awake humans, the nervous system can be accessed through recordings of motor unit action potentials. The motor unit (alpha motoneuron and all muscle fibres innervated by its axons), also known as the final common pathway of neural activation signals (Liddell and Sherrington 1925), is the transducer of synaptic sensory and descending inputs transmitted to the motoneuron pools into mechanical muscle actions (Heckman and Enoka 2012). The characteristics of motor unit action potentials, i.e., summation and time-course, determine the electromyogram (EMG; Farina et al. 2014; Enoka and Duchateau 2015), which can be recorded through the surface of the skin over the muscle (surface recordings) or from within the muscle (needle/fine-wire recordings; Adrian and Bronk 1928). With the existence of the high safety factor of transmission at the neuromuscular junction (Wood and Slater 2001), recordings of motor unit action potentials through EMG infer the discharges of individual motoneurons (Duchateau and Enoka 2011), making them the only nerve cells that can be recorded non-invasively in humans (Heckman and Enoka 2012). Initial studies investigating the compound (interference) EMG signal showed an increased amplitude in the early stages of resistance training concomitantly with increased muscle force generating capacity, suggesting neural contribution (Moritani and DeVries 1979; Häkkinen et al. 1998). However, the interference EMG amplitude is only a crude indicator of the neural drive to skeletal muscle (Farina et al. 2014; Del Vecchio et al. 2017), which precluded robust conclusion about the nature of adaptations. Subsequently, experiments employing advanced EMG recordings [e.g., intra-muscular or high-density surface EMG (HDsEMG)] and decomposition, that allow precise identification of motor unit discharge times, demonstrated that increased force production following resistance training is accompanied by decreased motor unit recruitment threshold and increased discharge rate (Van Cutsem et al. 1998; Kamen and Knight 2004; Vila-Chã et al. 2010; Del Vecchio et al. 2019), providing direct evidence of neural adaptations to resistance training at the level of individual motoneurons.

**Fig. 1:**
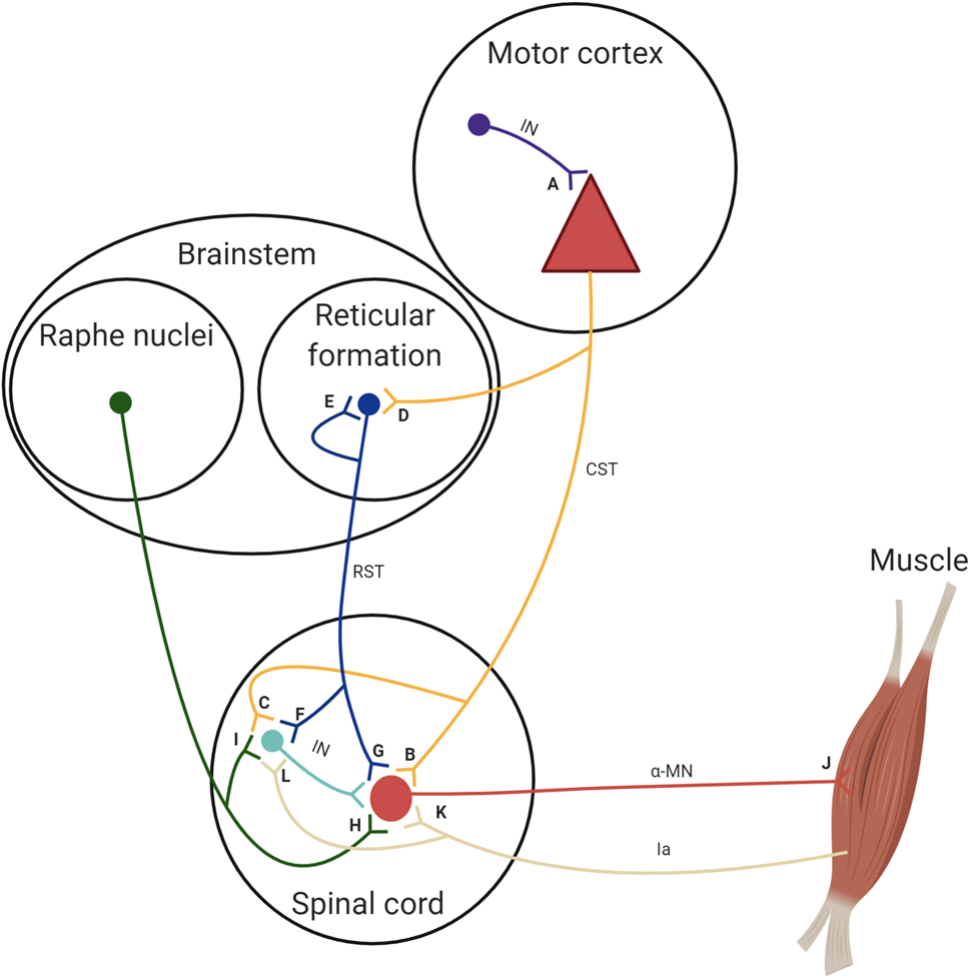
Possible sites of neural adaptation to resistance training. Many potential sites of neural adaptations to resistance training have been suggested. Changes in intracortical inhibitory interneurons (IN; A) have been demonstrated following resistance training in both human (Weier et al. 2012) and non-human primates (Glover and Baker 2020). Adaptations within the corticospinal tract (CST), the main conduit of movement signals in humans, have been equivocal, but may occur at the level of the corticomotoneuronal synapse (B) or via corticospinal projections to interneurons (C) (Nuzzo et al. 2017; Colomer-Poveda et al. 2019; Siddique et al. 2020). Though human data is lacking, experiments in primates suggest contribution of the reticulospinal tract (RST), a bilateral descending tract implicated in gross motor tasks, to increased force production following resistance training (Glover and Baker 2020), which may occur via corticoreticular connections (D), reciprocal reticular connections (E), reticulospinal projections to interneurons (F), or monosynaptic reticular projections to motoneurons (α-MNs; G). The potential neural substrate for resistance training adaptations is also the increased monoaminergic drive via brainstem projections, increasing the strength of persistent inward currents within motoneurons and thus up-regulating depolarisation and shortening the afterhyperpolarisation phase of motoneurons (H and I). Electrophysiological adaptations are also possible within the motor units themselves (J) and might be particularly potent in high-threshold motor units (Casolo et al. 2019). Finally, adjustments in the sensory feedback from muscle via Ia afferent neurons may occur with resistance training (Aagaard et al. 2002; Durbaba et al. 2013) either through monosynaptic connections to motoneurons (K) or via spinal interneurons (L). Adapted from Glover and Baker (2020)

Despite a clear demonstration of adaptation in the final pathway of the nervous system, the precise site of early neural adaptation causing changes in motor unit activity that accompany increased muscle force generating capacity following short-term resistance training remains elusive, with several mechanisms proposed (Fig. 1). Understanding the aetiology of neural adaptations is a critical consideration for the use of resistance training as a rehabilitation strategy for clinical populations, e.g., following stroke (Kim et al. 2019b), and for optimising resistance training programmes in athletes. Therefore, the purpose of this review is to clarify and critically discuss the literature concerning the site(s) of putative neural adaptations to short-term resistance training when morphological adaptations are expected to be largely absent. Particular attention will be given to the constraints of the current methodology to elucidate the site of neural adaptations, with suggestions for future investigations. Given the focus of the review is on the mechanisms underpinning the phenomenon of *increased force production* following resistance training, the review will be largely concentrated on studies employing single-joint/single-muscle isometric contractions during assessments, which allow the level of experimental control needed to isolate specific site(s) of neural adaptations. Regarding training interventions used throughout the literature, the studies included in the present review used similar training intensities (i.e., 75–80% 1RM) and volumes (i.e., 6–12 reps, 4 sets). Single-joint, isometric resistance training is the most commonly employed training modality (e.g., Nuzzo et al. 2017; Casolo et al. 2019; Del Vecchio et al. 2019), though multi-joint dynamic and ballistic interventions have also been assessed (e.g., Schubert et al. 2008; Weier et al. 2012; Ansdell et al. 2020). Whilst it is recognised that these factors may influence both the degree and mechanisms of neural adaptation, there is limited evidence systematically comparing the effects of these factors on neural adaptations to strength training, and such a discussion would be beyond the scope of the review. The fact that the majority of mechanistic studies reviewed in this article rely on simplified models of resistance exercise highlights the difficulty in obtaining neurophysiological data in response to more ecologically valid modes of resistance training (e.g., dynamic, compound resistance training), where methodological constraints challenge the ability to capture such data in a task-specific manner (Brownstein et al. 2018; Ansdell et al. 2020). The aforementioned factors should thus be considered when extrapolating findings from mechanistic studies into applied practice.

## Cortical or spinal adaptations: stimulation studies reveal inconsistent results

**Fig. 2:**
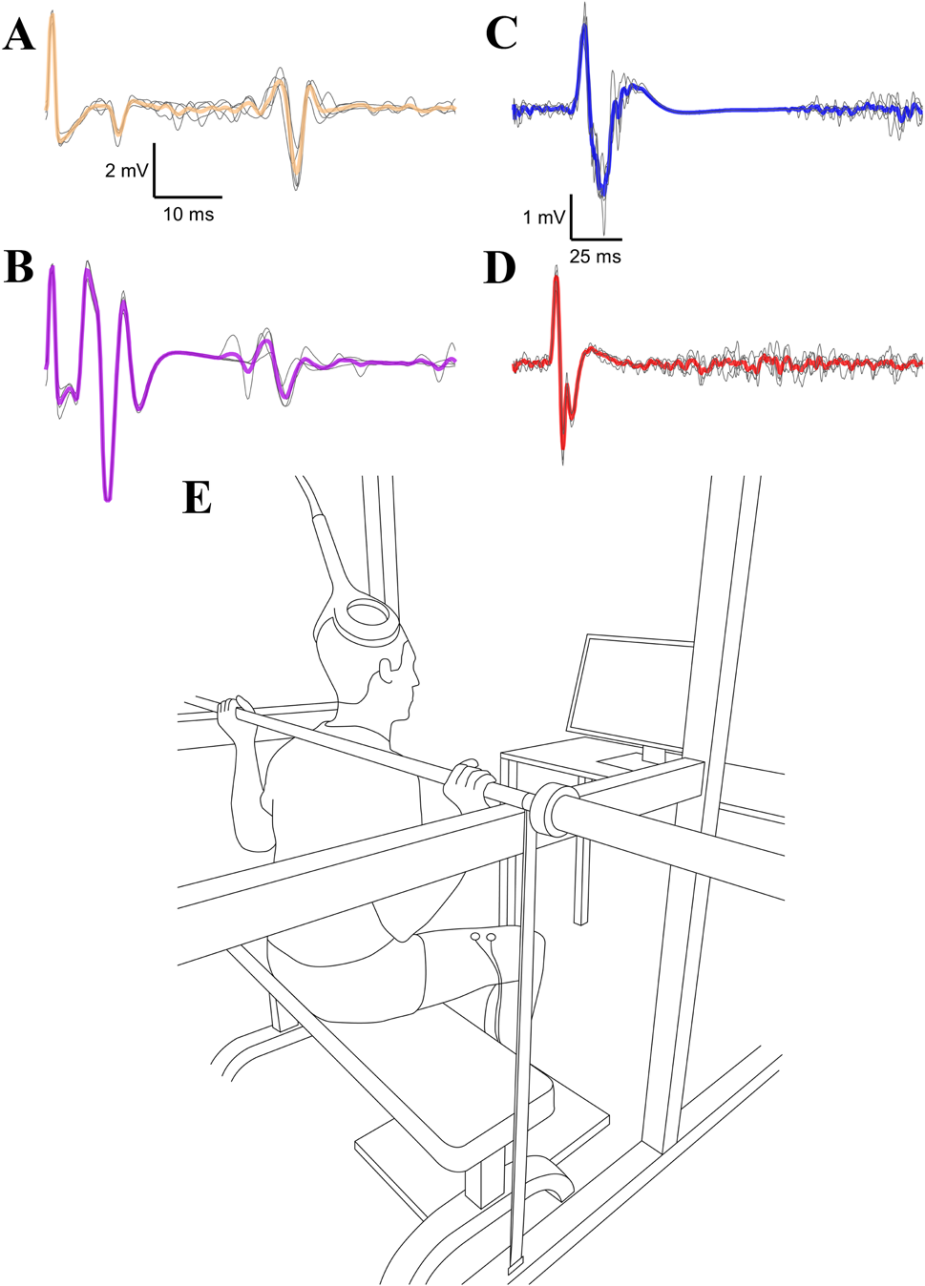
The responses commonly used to assess the site of neural adaptation to resistance training. Early studies have shown increased responses to percutaneous mixed nerve stimulation during a contraction following resistance training (e.g., Sale et al. 1983; Aagaard et al. 2002)—these responses are known as the H-reflex (**a**), which is a long-latency response to submaximal nerve stimulation often evoked with a small M-wave (note the short-latency response), and the V-wave (**b**), which is a long-latency response to supramaximal nerve stimulation (hence the presence of a short-latency maximal M-wave; for further details on methodology see Burke and Gandevia 1999). In recent decades, transcranial magnetic stimulation (for details on methodology see Rossini et al. 2015) has been used to infer the site of neural adaptation to resistance training; however, the response to such stimuli, known as the motor evoked potentials followed by the silent period (**c**), have yielded variable results when assessed after resistance training (for meta-analysis see Siddique et al. 2020). Since responses to transcranial magnetic stimulation alone cannot differentiate between the cortical and spinal site of adaptation, additional methods have had to be employed, such as responses to direct activation of corticospinal axons, e.g., lumbar evoked potentials (**d**), but they have been shown not to change following resistance training (Nuzzo et al. 2017; Ansdell et al. 2020). It is important to note that changes in responses to stimulation techniques following resistance training are likely to be specific to the training task (Kalmar 2018)—as a result there have been recent attempts to replicate the training task when assessing responses to stimulation (E; from Brownstein et al. 2018*, *with permission). Data displaying responses is from the personal archive of the authors—the average of 5 responses is displayed in colour with individual response overlaid in black

The early studies attempting to discern the site of neural adaptations to strength training employed stimulation of peripheral nerves and the study of reflex responses. The most commonly studied responses in the context of resistance training are the Hoffmann (H) reflex (Fig. 2a) and the V-wave (Fig. 2b), both of which involve stimulation of a mixed nerve and principally examine the monosynaptic spinal circuitry between the Ia afferent and alpha motoneuron (Fig. 1K). The H-reflex and V-wave differ in that the former is elicited with submaximal stimulation, whereas the latter is evoked with a supramaximal stimulation during a voluntary contraction (McNeil et al. 2013). Following resistance training, studies have generally shown no changes in the H-reflex amplitude when elicited at rest (Aagaard et al. 2002; Scaglioni et al. 2002), but an increase during strong contractions (Aagaard et al. 2002; Duclay et al. 2008; Schubert et al. 2008), consistent with the notion of task-specificity of neural adaptations. Similarly, V-waves, that can only be elicited during a contraction, have typically been shown to increase following resistance training (Sale et al. 1983; Aagaard et al. 2002; Fimland et al. 2009).

The authors of the aforementioned studies have often inferred that neural adaptations to resistance training are mediated at the ‘spinal’ level. However, the term ‘spinal’ is rather broad, and both H-reflex and V-wave are subject to technical limitations that preclude identification of the precise site of neural adaptation (for reviews see Burke and Gandevia 1999; Zehr 2002; Knikou 2008). Briefly, due to activation of Ia afferents, the H-reflex is subject to modulation of presynaptic inhibition (for review see Zehr 2002). Thus, increased H-reflex amplitude following resistance training (Aagaard et al. 2002) may be primarily associated to downregulated presynaptic inhibition of sensory inputs rather than adaptations within the motor pathway. Furthermore, H-reflex is sensitive to changes in axonal excitability (Bostock and Grafe 1985), meaning that increased amplitude of the reflex may not necessarily represent changes within the spinal cord. Though less likely to influence the V-wave (Burke and Gandevia 1999), changes in axonal excitability represent a potential confound regardless of whether one attempts to ensure a similar proportion of motoneuron pool activation when eliciting the H-reflex (e.g., standardised M-wave preceding H-reflex; Zehr 2002). Finally, both the H-reflex and V-wave have non-monosynaptic contributions (Fig. 1L; Burke et al. 1984; Marchand-Pauvert et al. 2002); thus, the changes in reflex response could be the result of alterations in synaptic efficacy in one or both of these circuits (Burke and Gandevia 1999). In essence, changes in H-reflex and V-wave do not necessarily measure motoneuron excitability as the latter typically assumes a predominantly monosynaptic contribution (McNeil et al. 2013).

The advent of transcranial magnetic stimulation (TMS; Barker et al. 1985), which allows non-invasive, painless activation of neurons within the motor cortex (for review see Rossini et al. 2015), has resulted in a proliferation of studies investigating motor control and neural alterations to various motor tasks, including resistance training (Carroll et al. 2011; Kidgell and Pearce 2011). The size of the response to TMS, the motor evoked potential (Fig. 2c), can be recorded at the muscle via EMG and represents an index of excitability of the corticospinal tract (see CST in Fig. 1, Bestmann and Krakauer 2015). Perhaps the most consistent experimental observation using TMS is that the motor cortical inhibition is decreased with resistance training (Fig. 1A; Goodwill et al. 2012; Weier et al. 2012; Leung et al. 2017), though contradicting evidence does exist (Beck et al. 2007; Ansdell et al. 2020). The majority of these studies utilised similar training intensities and volumes (75–80% 1RM, 6–12 repetitions, 4 sets; Goodwill et al. 2012; Weier et al. 2012; Leung et al. 2017; Ansdell et al. 2020), though other characteristics of the training differed (e.g., unilateral, Goodwill et al. 2012; Leung et al. 2017; bilateral, Weier et al. 2012; Ansdell et al. 2020; lower limb, Goodwill et al. 2012; Weier et al. 2012; Ansdell et al. 2020; upper limb, Leung et al. 2017), possibly explaining the discrepant results.

The activity of inhibitory interneurons in the motor cortex is principally assessed with paired-pulse TMS, whereby two pulses are delivered with a short interstimulus interval, and the responses are thought to be underpinned by the activity of receptors of gamma aminobutyric acid (Ziemann et al. 1996; Di Lazzaro et al. 2007), the main inhibitory neurotransmitter in the human central nervous system. This neurotransmitter has been heavily implicated in motor learning (Bachtiar and Stagg 2014), which supports the notion proposed a few decades ago that resistance training is a form of motor leaning (Sale 1988). Thus, one potential argument is that the neural adaptations and increased strength observed in the initial stages of resistance training may reflect the processes implicated in motor learning. In addition to paired-pulse responses to TMS, the duration of the silent period following the evoked response to TMS has also been shown to be reduced following resistance training (Christie and Kamen 2014; Siddique et al. 2020) and used to infer a reduction in the activity of intracortical interneurons; however, this interpretation of the phenomenon has been questioned, with suggestions that adjustments within the spinal network could be complicit (Yacyshyn et al. 2016; Škarabot et al. 2019b). Nevertheless, meta-analyses generally support the premise that resistance training alters excitability of the intracortical inhibitory interneurons, particularly when these are assessed during a voluntary contraction (Kidgell et al. 2017; Siddique et al. 2020). Similar reductions in intra-cortical inhibition have been demonstrated following acute aerobic exercise (Singh and Staines 2015; El-Sayes et al. 2019), perhaps suggestive of a mechanism linked to exercise in general, rather than specific to resistance training.

Evidence of changes in corticospinal excitability has been inconsistent, with an increase (Griffin and Cafarelli 2007; Weier et al. 2012), decrease (Carroll et al. 2002; Beck et al. 2007; Giboin et al. 2018) or no change (Carroll et al. 2009; Christie and Kamen 2014; Coombs et al. 2016) shown following short-term resistance training. The differences in experimental designs, particularly as they relate to the training protocol, and the lack of agreement between the training and testing task (Avela and Gruber 2011; Kalmar 2018) likely contribute to these discrepancies. For example, whilst the majority of studies employed training intensities between 70 and 100% maximum intensity, these were a mixture of isometric (Griffin and Cafarelli 2007; Christie and Kamen 2014; Giboin et al. 2018) and dynamic contractions (Carroll et al. 2002; Beck et al. 2007; Weier et al. 2012; Coombs et al. 2016). When these factors are coupled with inconsistent methodological approaches to measure indices of corticospinal excitability and/or inhibition, the equivocal nature of the literature is perhaps unsurprising. The mechanistic extrapolation is further complicated, because whilst TMS activates pyramidal neurons in the motor cortex through indirect activation, the response measured in the EMG activity (motor evoked potential) represents inputs from both cortical as well as spinal centres (Rossini et al. 2015). Indeed, changes in motor evoked potentials following resistance training could represent alterations within the motor cortex itself, within the spinal cord, or in the efficacy of the synapses leading to the motoneuron (e.g., Fig. 1B, C). Additional methods, such as assessing responses to direct activation of corticospinal axons at subcortical levels (Fig. 2d; Taylor and Gandevia 2004; Martin et al. 2008; Škarabot et al. 2019a) is required in conjunction with TMS to make a distinction as to whether the site of neural adaptations lies within the motor cortex or subcortically. Few studies have examined responses to direct activation of corticospinal axons following short-term resistance training, but their findings agree that neural adaptations are not mediated by intrinsic changes to motoneurons, efficacy of corticomotoneuronal synapses or transmission efficacy along descending pathways (Nuzzo et al. 2017; Ansdell et al. 2020).

Taken together, the general inconsistencies in the literature on the site of neural adaptations to resistance training inferred from stimulation techniques are widespread. However, the equivocal nature of findings from studies employing TMS does not necessarily exclude the implication of the motor cortex and/or the corticospinal tract in neural adaptations to resistance training. A consideration of the limitations of techniques used to study corticospinal changes following resistance training, as well as the context of their use, might provide explanations for equivocal results or offer alternative considerations. Firstly, the relationship between TMS-induced responses and behavioural outcomes is complex and not always directly interrelated. Indeed, TMS may activate elements of the motor output that are not necessarily directly related to volitional neural activity. For example, TMS responses provide information about the population of neurons activated by stimulation, which represent presynaptic interneural inputs and postsynaptic corticospinal excitability, which may not be directly relevant to motor behaviour (Bestmann and Krakauer 2015). Other technologies that permit inferences of central nervous system behaviour during volitional actions could overcome this limitation, as discussed in subsequent sections. Additionally, changes might occur in cortical areas outside the primary motor cortex, which may or may not cause changes in the population of neurons activated by stimulation (Bestmann and Krakauer 2015). Secondly, responses to stimulation techniques are known to be variable; this has been suggested to be due to inter-individual variability in synaptic efficacy of different neuronal populations and subtle changes in electrophysiological properties of neuronal populations within an individual (Orth et al. 2003). Because of this variability, methodological nuances can influence the sensitivity to detect changes, especially if these are subtle. Thirdly, the training and the assessment tasks typically differ in the generation of the motor command, which can mask potential changes in neural responses. For example, even when attempts have been made to replicate biomechanical characteristics of the training task when measuring TMS-induced responses (Fig. 2e; Brownstein et al. 2018), recordings were made during a low-intensity isometric contraction, which differed considerably to the training task involving dynamic squats with 80% of 1-repetition maximum (Ansdell et al. 2020). However, when the intent to produce force, and thus likely the motor command, was replicated in the assessment task motor evoked potentials showed a clear task-specific change (Giboin et al. 2018). Future studies could consider investigation of TMS-induced responses during the movement preparation phase, which represents an experimental lens into the motor command (Tanji and Evarts 1976; Cisek and Kalaska 2010). Finally, although the corticospinal tract represents the primary pathway controlling skeletal muscle, it is possible that the main site of neural adaptation lies outside the direct corticomotoneuronal connection. Other descending tracts could be considered sites of neural adaptation to resistance training, such as the reticulospinal tract. Several characteristics of the reticulospinal tract provide a rationale for its implication in the neural causes of strength increase: its bilateral nature could facilitate certain exercises (Jankowska et al. 2003); its collateralisation could enable the activation of muscle synergies during gross motor tasks (Peterson et al. 1975); as well as its direct and indirect (via an interneuron) projections with motoneurons (Riddle et al. 2009). Furthermore, when lesions were made in the pyramidal (Lawrence and Kuypers 1968) and corticospinal tract (Zaaimi et al. 2012, 2018) of non-human primates, a ‘compensatory’ increase in the efficacy of reticulospinal connections with the motoneuron was observed that accompanied the recovery of strength. These findings provide the neuroanatomical, neurophysiological and behavioural basis that make the reticulospinal tract a potent substrate for neural adaptations to resistance training. Indeed, direct stimulation of the reticulospinal tract in non-human primates reveals increased responses following resistance training suggesting adaptation in this tract, likely through monosynaptic (Fig. 1H) and disynaptic (via an interneuron; Fig. 1I) connections to motoneurons, decreased corticoreticular connection (Fig. 1D) and/or increased reciprocal reticular connection (Fig. 1E; Glover and Baker 2020). Though reticulospinal tract function is not possible to assess directly in humans, startle reaction time tasks (Baker and Perez 2017) and auditory startle cues combined with TMS (Tazoe and Perez 2017) and transcranial electrical stimulation (Furubayashi et al. 2000) have been used previously to infer reticulospinal function in humans and might be worth considering in future studies investigating neural adaptations to resistance training.

The range of potential adaptation aetiologies means that relying on TMS or other non-invasive neurostimulation paradigms alone might limit the inferences that can be made by a single experiment. As will be discussed in the next sections, technologies that allow inferences to be made regarding central nervous system behaviour during volitional actions might provide routes for further exploration of neural adaptation to resistance training.

## High-density surface electromyography: potential for source identification

**Fig. 3:**
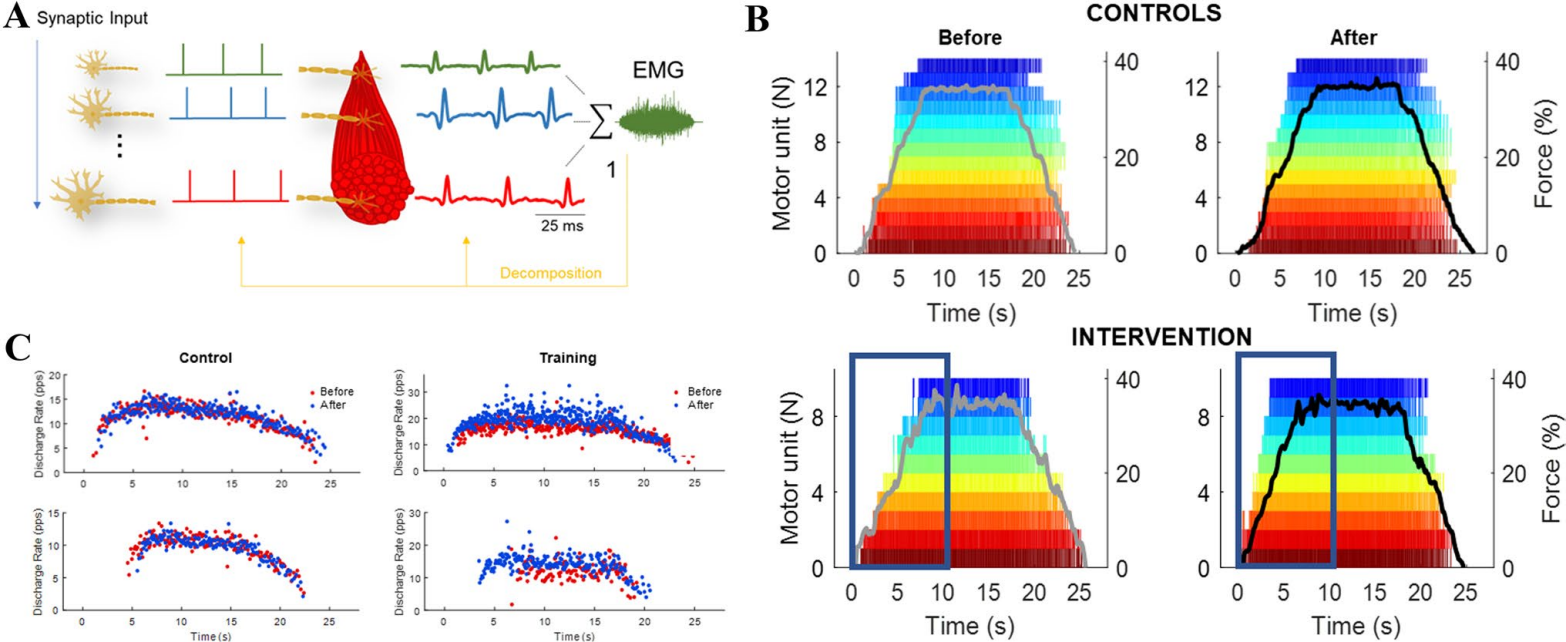
Motor unit changes following strength training. **a** Whilst the more invasive fine-wire/needle electromyography is still considered the ‘gold standard’ for discerning the activity of single motor unit action potentials, recent advances in technology have allowed decomposition (line 1 in orange) of the interference electromyogram (the summated motor unit activity) from surface recordings (i.e., high-density EMG). Inferring changes in the nervous system from the global surface EMG is limited due to amplitude cancellation and the non-linear relationship between the size of action potentials and recruitment threshold; however, decomposition of the signal into individual motor unit spike trains infers activity of single motoneurons due to one-to-one relationship between axonal (left) and motor unit (right) action potentials by the muscle unit. From Del Vecchio et al. (2020)*,* with permission. **b** A raster plot of decomposed motor unit spike trains from high-density EMG during a trapezoidal contraction at 35% of maximal force before and after short-term resistance training (intervention) or no change in physical activity (control). Short-term resistance training decreased motor unit recruitment thresholds (note the dark blue boxes), whereas derecruitment thresholds remained unchanged. Adapted from Del Vecchio et al. (2019)*, *with permission*.* **c** Concomitantly with decreased recruitment thresholds, motor unit firing rate have also been shown to be augmented with short-term resistance training when the same motor units are tracked across time (Del Vecchio et al. 2019), whereas no such phenomenon is observed in the control group; consistent with the data previously obtained from fine-wire electromyography (Van Cutsem et al. 1998). The scatter plot and data from Del Vecchio et al. (2019)*, *with permission

Stimulation techniques, including TMS, involve the study of evoked responses. Therefore, stimulation methods will always be, to some extent, limited in their ability to make inferences about behavioural outcomes as they do not allow capturing changes in volitional neural activity. On the other hand, recent technological advances allow a non-invasive study of the activity of large populations of motor units during voluntary contractions in the full recruitment range of a muscle through careful decomposition of HDsEMG (Fig. 3a; Holobar and Zazula 2007; Farina et al. 2016; Del Vecchio et al. 2020). Furthermore, due to the spatial ‘signature’ of each motor unit discharge, it is possible to longitudinally track motor units across recording sessions (Martinez-Valdes et al. 2017; Del Vecchio and Farina 2020), thus allowing direct comparison of potential changes in motor unit properties as a result of an intervention (e.g., training/rehabilitation protocol). Using this methodology, it has recently been shown that increased force production following short-term resistance training was accompanied by decreased recruitment threshold (Fig. 3b) and increased firing rate (Fig. 3c) of a large population of longitudinally-tracked motor units (Del Vecchio et al. 2019). These findings clearly support that early adaptations to resistance training are of neural origin. However, since motor unit activity represents transformation of synaptic sensory and descending inputs, establishing the origin of decreased recruitment and augmented firing rate is challenging.

It is important to note that motoneurons receive two types of inputs; ionotropic, which depolarise and hyperpolarise motoneurons, sub-serving specific motor commands and reflexes (Heckman and Enoka 2012), and neuromodulatory, which involve binding of second-messenger systems (e.g., serotonin, noradrenaline) released by the axons of the brainstem raphe nuclei that bind on G-proteins and activate voltage-dependent channels on the motoneuron dendrites (Fig. 1H, I; Heckman and Enoka 2012). The latter allows the generation of strong persistent inward currents, which can increase responsiveness of motoneurons to ionotropic inputs (Heckmann et al. 2005). It is possible to estimate the strength of persistent inward currents in humans with the so-called paired motor unit technique during voluntary contractions with a prescribed trajectory of force increase and decrease (Gorassini et al. 2002; Afsharipour et al. 2020). Specifically, during the ascending phase of the contraction a relatively low-threshold (control) motor unit increases its firing frequency whilst a second, higher-threshold (test) motor unit is recruited, which then continues firing during the descending phase of the contraction at lower levels of synaptic input required to recruit it in the first instance. The strength of the persistent inward currents is then estimated as motor unit recruitment hysteresis, which is quantified as the difference between the instantaneous firing frequency of the control unit at test unit recruitment and derecruitment. Alternatively, motor unit saturation has also been suggested as a potential estimate of persistent inward current strength (Johnson et al. 2017) as it appears to be inherently linked to neuromodulatory input (Hyngstrom et al. 2008; Revill and Fuglevand 2017). Motoneuron afterhyperpolarisation duration has been shown to decrease following short-term resistance training (Christie and Kamen 2010), which might indicate increased flow of positive charged ions onto the motoneurons and thus increased probability of action potential generation, possibly as a result of increased monoaminergic drive. Furthermore, in the study by Del Vecchio et al. (2019) recruitment threshold of motor units were found to be decreased, but no changes were noted in derecruitment threshold relative to force produced, suggesting the hysteresis of motor unit recruitment had changed as a result of resistance training (Kim et al. 2019a). However, the lack of changes in the motoneuron input–output relationship (the relationship between motor unit discharge rate and force production) cast doubt that increased neuromodulatory input contributed to increased force production following short-term resistance training (Del Vecchio et al. 2019). Nevertheless, a more direct investigation into the role of neuromodulatory inputs to motoneurons following resistance training is warranted, particularly since data on rodents suggest that alterations in ionic conductance of motoneurons and augmented electrophysiological properties of both slow and fast-type motoneurons are evident after resistance training (Gardiner et al. 2006; Krutki et al. 2017).

Due to lack of changes in the motoneuron input–output relationship following resistance training, decreased motor unit recruitment and augmented discharge rate are likely of supraspinal origin (Del Vecchio et al. 2019). However, as already discussed, data from stimulation studies is inconclusive concerning the role of the motor cortex in the adaptations to resistance training and is limited insofar as it does not provide information about volitional muscle activity. Cortical activity underpinning volitional muscle activity can be assessed using electroencephalography (EEG), which measures postsynaptic brain activity with high temporal resolution. The negative excitatory post-synaptic potentials in EEG around the time of voluntary movement, known as movement-related cortical potentials, have been shown to display attenuated amplitude at several scalp sites during the same relative force levels following resistance training (Falvo et al. 2010). Furthermore, recent data in non-human primates has indicated a supraspinal contribution to resistance training adaptations (Glover and Baker 2020). These findings imply that the motor cortical demand is reduced with increased force production as a result of resistance training. However, it is unclear whether motor cortical demand is solely reduced, or whether it reflects changes in reticulospinal and/or intraneuronal networks projecting to spinal motoneurons. Pairing EEG with HDsEMG recordings and analysing the coherence between cortical and motoneuronal signals in specific frequency domains (Gallego et al. 2015; Holobar et al. 2018) may facilitate such understanding.

Finally, it is important to highlight that presently available data suggest motor unit adaptations following resistance training are not threshold-specific (Van Cutsem et al. 1998; Del Vecchio et al. 2019). Following short-term resistance training, motor unit conduction velocity has been shown to increase selectively for high-threshold motor units (Casolo et al. 2019); however, this likely reflects changes in electrophysiological properties of muscle fibres (e.g., alterations in the capacity and transport activity of NA^+^-K^+^ pump), rather than alterations in neural synaptic input. The uniform increase in discharge rate across the motor pool is also inconsistent with the idea of augmented reticulospinal input following resistance training (Glover and Baker 2020), since this tract seem to preferentially recruit higher-threshold, larger motoneurons (Ziemann et al. 1999). However, different inputs could be augmented concurrently with resistance training; for example, reticulospinal input that may have a bias towards higher-threshold motoneurons, neuromodulatory input that is longer-lasting in low-threshold motoneurons (Lee and Heckman 1998), with possible additional inputs from interneural networks in the motor cortex. Future research should thus consider concomitant contribution from different sources of input that are likely responsible for uniform changes in motoneuron discharge rate across the entire motor pool.

## Further considerations and conclusions

The present review has principally discussed neural adaptations to resistance training based on recordings of the agonist muscle(s). Indeed, the literature has predominantly focused on neurophysiological changes in the agonist muscles, with relatively little regard for antagonist and synergists. However, increased force production of the agonist muscle following resistance training may occur due to upregulation of activity within the agonist itself, as well as suppression of the antagonist and/or facilitation of synergist muscles. Early studies investigating interference EMG amplitude suggest reduced antagonist activation following resistance training (Carolan and Cafarelli 1992), though conflicting evidence also exists (Holtermann et al. 2005). Notably, muscles are not controlled by distinct territories within the motor cortex, but are overlapped and intertwined, and more likely interconnected by intrinsic collaterals involved in the integrated control of muscle synergies (Devanne et al. 2006; Capaday et al. 2013). Thus, it is conceivable that focusing on recordings of a single, typically the agonist muscle neglects the possibility of changes in intermuscular coordination as a result of resistance training. Whilst coordination is conceptually difficult to measure with stimulation techniques such as TMS, the distribution of different inputs to the motoneuron pool between synergists has been investigated previously (Laine et al. 2015), but not in the context of resistance training. Future studies should consider concomitant recordings of synergists and antagonists to provide a broader understanding of neural adaptations to resistance training within the whole motor pool.

In conclusion, there is considerable evidence consistent with the notion that the early increases in force production following resistance training are underpinned by neural adaptations. However, despite the proliferation of studies in the field in the last two decades, the precise site of putative neural adaptations remains unclear. Based on the available evidence, it is likely that neural adaptations are underpinned by alterations in the cortical and/or subcortical structures, with changes in inhibitory cortical interneurons and reticular formation being the most potent candidates. The advances in decomposition of neural signals (i.e., HDsEMG), coupled with the use of existing methods (e.g., EEG), as well as indirect probing of reticular formation in humans (e.g., via auditory startle stimuli), have considerable potential to contribute to completing the puzzle regarding the site of neural adaptations to resistance training in the coming decade.

## Abbreviations

EEG: Electroencephalogram
EMG: Electromyogram
HDsEMG: High-density surface electromyogram
H-reflex: The Hoffman reflex
TMS: Transcranial magnetic stimulation
